# A Threat of Customer Incivility and Job Stress to Hotel Employee Retention: Do Supervisor and Co-Worker Supports Reduce Turnover Rates?

**DOI:** 10.3390/ijerph18126616

**Published:** 2021-06-19

**Authors:** Hyunah Chung, Wei Quan, Bonhak Koo, Antonio Ariza-Montes, Alejandro Vega-Muñoz, Gabriele Giorgi, Heesup Han

**Affiliations:** 1 College of Hospitality and Tourism Management, Sejong University, 98 Gunja-Dong, Gwanjin-Gu, Seoul 143-747, Korea; hyunah0124@naver.com (H.C.); rj782615@gmail.com (W.Q.); 2 School of Hospitality and Tourism Management, Spears School of Business, Oklahoma State University, 365 Nancy Randolph Davis Building, Stillwater, OK 74078, USA; bkoo@okstate.edu; 3 Social Matters Research Group, Universidad Loyola Andalucía, C/Escritor Castilla Aguayo, 4 14004 Córdoba, Spain; ariza@uloyola.es; 4 Public Policy Observatory, Universidad Autónoma de Chile, 7500912 Santiago, Chile; alejandro.vega@uautonoma.cl; 5 Department of Human Sciences, Università Europea di Roma, 00163 Roma, Italy; gabriele.giorgi@unier.it

**Keywords:** job stress, turnover, customer incivility, perceived supervisor support, perceived co-worker support

## Abstract

The study investigates the impact of customer incivility, job stress, perceived supervisor support, and perceived co-worker support on the turnover intention of frontline employees. A survey-questionnaire approach was used to collect the point of view of frontline employees that work in five-star hotels in a metropolitan city of Korea. Four independent variables that were extracted from valid theoretical backgrounds along with four demographic variables were used in the study. The regression analysis was conducted to test the hypotheses, which revealed that job stress directly affected the employees’ desires to leave their organization. It also showed that perceived supervisor support mitigates employee turnover, and there were significant correlations between turnover intention with the employees’ marital status and job position. Gender and years of service did not affect the employees’ thinking of quitting their job. Our findings help hotel entrepreneurs better understand how to deal with customer incivility and employee job stress, and better comprehend the factors that minimize employees’ negative behaviors for the organization.

## 1. Introduction

The customers have always been the most important factor in the hospitality sector [1] and companies have never stopped their effort to learn the needs and the trends of its consumers’ satisfaction. In 2019, 1.5 billion international tourist arrivals were recorded, and a 3–4% increase was predicted for 2020 before the outbreak of the COVID-19 pandemic, which still marks tourism as a leading economic sector [2]. As the competition within the tourism and hospitality industry become severe, it is indisputable that customer demands are also increasing [3].

The power and the influence consumers have has also escalated due to the spread of social media [4]. Consumers look for personal comments from other previous consumers in the decision-making process regarding consuming almost everything. One customer’s personal comment could be delivered to neighbors as well as unspecified customers all over the world, which can promote dramatic sales but can also be an emotional and retaliatory occurrence that brings burdensome prices to pay for the companies [5]. Considering the unique culture of the hotel industry where customers expect high quality not only with physical products they purchase but also in the way they are presented and delivered to them as an experience, it is never enough to highlight the importance of the frontline employees (FLEs) in the hotel industry who deliver essential services to customers to elevate satisfaction and loyalty [6,7,8,9,10].

In order to provide consistent high-quality products and services, retaining skillful and customer-oriented FLEs who are able to persistently evaluate all the circumstances, identifying the customer needs with the help of knowing each customer’s preferences and being able to satisfy their needs are crucial for organizations [8,11]. Even though various empirical studies have been conducted that analyze these factors e.g., [9,12,13], the effect of each of these factors evolves as society, customer behavior, and the pursuit of the employees change over time. For instance, factors, such as low wages and long working hours that were pointed out as the causes of the turnovers in the past have been mitigated by government laws. Customer incivility, which is unreasonable behaviors and demands of the customers are still part of the consumer’s role in the exchange situation [14], but some actions diminish as society becomes more mature and civilized. Therefore, investment to cultivate employee satisfaction and reduce employee turnover is essential to implement the standards for quality customer service [9].

Therefore, the main purpose of this study is to explore the relationships between the turnover intention of hotel FLEs in five-star hotels that are located in Seoul and the major factors that are taken into academic consideration. The study aimed to examine the influence of customer incivility, job stress, social support on the employee turnover intention, and how the demographic features, such as gender, marital status, years of service, and job position influence turnover intention.

## 2. Literature Review

### 2.1. Turnover Intention

While some researchers are aware of the positive aspects of employee turnover [15,16], the employee turnover is mostly recognized as a negative term due to the effort that is required for recruiting, the training expenses that organizations have to pay to replace the vacancy, and the negative impact on customer service [9,17,18,19]. The previous studies have shown that the turnover rate costs in the hospitality industry, which might vary by region and culture, is exceptionally high resulting in the loss of revenue up to 15 million US Dollars [20,21].

The turnovers can be divided into organizational turnovers and occupational turnovers, but they have similar effects with the way they make institutions confront additional problems they did not have to necessarily face [22]. A rather important classification of turnovers within the organizations was presented by Wanous, Stumpf, and Bedrosian [23], which classified turnovers into two categories, voluntary turnovers, and involuntary turnovers. Voluntary turnovers are turnovers that are avoidable, which are caused by factors such as the salary, benefits, workplace stress, relationships with other staff, and the working hours, whereas involuntary turnovers are turnovers that are unavoidable due to contract expiration, retirement, illness, or death [18,23]. Involuntary turnovers are aroused by the inevitable factors that do not consider the individual’s intent, so the employee turnover intention, meaning the employee’s “conscious and deliberate willfulness to leave a certain position in the organization after a certain period of time” [24,25], is used to represent the intentions of only the voluntary turnovers in this study.

Employees in the hospitality industry, especially in the hotel sector, have endured a particular working environment that is different from other working environments in most other business industries. Demanding service interactions, seasonal variations [26], scheduling, frequent workdays on national holidays, physical demands of the job, job insecurity especially from unexpected events (e.g., political issues or a pandemic, such as COVID-19 that has a direct influence on the tourism industry), have been pointed as unique but problematic elements of the industry that contribute to high turnover rates along with chronic issues, such as low remunerations and minimal promotion opportunities [9,21], which cause the employees to pursue alternative job opportunities or take a career break [27,28].

In this study, the turnover intention is used as a variable instead of the actual turnover rate, because just by having the intention to leave the organization could undermine the employee’s productivity, which again strongly relates to customer satisfaction especially for those who work as FLEs. In addition, employees generally feel the intent to quit the job before they actually make the decision to leave the organization, which means a high turnover intention will eventually be a possible repercussion for a high turnover rate. Finally, it is more practical to research the withdrawal intention of the employees who are still working in the industry rather than tracking down the employees who have already left the organization.

### 2.2. Customer Incivility

Recognition of the wasted cost and the negative impacts of customer misbehavior on both the businesses, their employees, and also on other customers have led to various empirical studies on these negative actions of the customers especially in the field of hospitality where various services are provided to its customers [1,3,6,29,30,31]. Customer service representatives are the frontline employees (FLEs) of the organizations, and these employees have to tolerate not only the stressors, which are common in other workplaces, but also the negative emotions and uncivil actions from their customers [7,32].

The term that refers to any incivilities that are shown from the customers varies considerably due to the long amount of time the concept has attracted scholarly attention in the field and the ways the behaviors are categorized differ between scholars. Bitner, Booms and Mohr [12] labeled problematic incidents that the employees encounter as *problem customer behavior* and categorized it into four groups, which include drunkenness, verbal and physical abuse, breaking company policies or laws, and uncooperative customers. Lovelock [33] used the term *jay-customers* to refer to the customers who “deliberately act in a thoughtless or abusive way” and defined six categories of jay-customers, which include thieves, deadbeats, vandals, rule breakers, belligerents, and family feuders. Fullerton and Punj [14] defined the consumer misbehavior as actions that are against the generally accepted norms made during the consumption process. Harris and Reynolds [34] extended the types to include compensation letter writers, undesirable customers, property abusers, service workers, vindictive customers, oral abusers, physical abusers, and sexual predators, which they later defined the various acts of those customers as “dysfunctional customer behavior” [1]. Berry and Seiders [5] emphasized that *unfair customers* should be considered independent of illegality, and Boo, Mattila, and Tan [35] categorized *deviant customer behavior* as grungy, inconsiderate, rule breaking, crude, violent or physical abusive, and verbal abusive.

Even though the categorizations of the earlier studies are still valid, the recent studies focused more on the customer actions the employees have to encounter during service interactions, which are legal yet deleterious [5,6,7,8,9,10,11,12,13,14,15,16,17,18,19,20,21,22,23,24,25,26,27,28,29,30,31,32,33,34,35,36]. A customer who stole a certain item from a property can be punished by the law, but customers who accumulate the complimentary items, which include amenities provided in hotels, free drinks, or extra ingredients that are set up freely for customer convenience, can be more stressful to organizations, employees, and other customers than the customers who have actually committed an illegal action. Verbal abuse with negative expressions shown by customers without any physical abuse can also be another example of incivility that employees have to endure.

How to evaluate these negative customer behaviors as uncivil or unfair was presented by Berry and Seiders [5] using three concepts. Customer behaviors that reach either moderate or intermittent level in terms of two factors, the severity of the damage the customer has created, and the *frequency* of the problematic customer behavior, are considered to have crossed the threshold of unfairness. Additionally, if the behaviors were intentionally committed to take advantage, harm others, or disrespect the rights of others, which include both employees and other customers, they should be considered unfair.

However, customer incivilities can occur even without knowing that they occurred, which is either due to the action being unintentional or because of the perpetrator’s lack of awareness [37]. One customer’s uncivil incident could be overlooked, but the effect of facing these types of behaviors repeatedly and frequently could be as detrimental as facing deviant and violent customer behaviors [32,36]. Therefore, the term customer incivility in this study will represent the repugnant customer behaviors that are categorized as legal actions as well as low-quality, interpersonal treatment, which makes it hard for both the company and the employees to respond accordingly [38].

### 2.3. Job Stress

Job stress can be interpreted as a condition of stress that is aroused by any of the stress factors that are correlated with work. There are various factors that cause job stress for employees within the working environment, such as employee’s capabilities, work tasks, job characteristics, the work place, relationships with co-workers, role conflicts, and heavy workloads [39,40]. In the beginning of the term’s conceptualization, it was defined as a state that is caused either by not being able to fulfill and meet the requested demands or not being able to achieve satisfactory amounts of supplies that are necessary [41]. Further studies have focused more on the psychological aspect and considered job stress as an emotional response of an individual to an excessive work load, which is a behavioral reaction that is drawn by role conflict and role ambiguity [42,43].

The importance of recognizing the job stress of employees has been explained in previous studies because they are closely related to the outcomes of an organization and the overall atmosphere of the job environment [44,45]. When the job stress feeling becomes severe with the employees, it negatively affects the company outcome, the service they provide to the customer, and it draws negative attitudinal and behavioral outcomes [44,46]. Job stress is also injurious to the employee themselves. It is associated with many physical and psychological health problems [47], such as hypertension, cardiovascular disease, immune disorders, obesity, and depression. Additionally, since work constitutes a major source of people’s daily lives [48], it may affect the employees’ intentions to withdraw from stress arousing environments for the sake of their own well-being [39,45,49,50,51].

The work characteristics of the hospitality industry especially for the FLEs can be regarded as unattractive, which cause sleep disorders and other health-related issues due to the antisocial working hours [45,52], frequent schedule changes, heavy volume of work during working hours along with long working hours, face to face interactions with a variety of guests, seasonality, low job security and wages, limited opportunities for personal development [52,53], and can become stressors for the employees in the hospitality industry, which can explain the high turnover rate that the industry faces [13].

### 2.4. Social Support

Social support is the norm that extensively consists of all the support an individual can achieve through interpersonal relationships and the quality of those relationships [54]. There are a variety of support types an employee can endure in order to ease the contribution of the work stressors [55]. From the context of social support, the perceived supervisor support is the degree that employees perceive the support and concern regarding job performances and the psychological and physical well-being of an employee in the working environment [56,57].

Supervisors are the people who are in charge of the employees’ performance and are responsible for the actions made by the employees, and the support provided from them can be divided into instrumental support, which offers help in a tangible way such as providing money, and emotional support, which is based on intangible assistance such as providing sympathy and recognition [58,59]. They have the power to influence the work environment and the status of the employees, which can impact the affective reactions of their team members that make supervisory support recognized as an important support entity in the workplace, and it has strong relationships with employees’ job satisfaction, job stress, job performance, and organizational commitment [55,57,58,60,61].

Perceived co-worker support is the degree that employees receive the support in the workplace relationships from their peers [55]. Hochschild [62] observed that employees face emotional dissonance because they have to express a feeling differently from the feeling they actually have. As a result, employees tend to form a community to share their true feelings with fellow co-workers in order to reduce stress. Having people around who can truly understand and sympathize with the incidents that occur in the workplace and the emotions that follow for both enjoyable and challenging encounters may mitigate the effects of stressors, and the employees that do not have any perceived co-worker support can suffer more from the effects of the work stressors. The researchers have also explored the role of co-worker support and proved that it would either significantly or insignificantly affect the employees’ job performance, satisfaction, and organizational commitment [55,63,64]. Susskind, Kacmar, and Borchgrevink [65] specified the term defining co-worker support as the extent of employees’ beliefs that their co-workers would help themselves with work-related issues or provide task-related information. Hochschild [62] emphasized the socioemotional assistance from co-workers, and Susskind et al. [65] illustrated the actual work-related support from co-workers.

For this study, the perceived supervisor support and the perceived co-worker support were separated from the social support in order to find the effect emotional support received interpersonally within the workplace has regarding the employees’ turnover intention. Additionally, this study tried to measure the perceived support that employees in the workplace receive, which relates to the personal emotional feelings that the employees can receive from their supervisor and co-worker.

### 2.5. Research Model and Hypotheses

#### 2.5.1. Customer Incivility and Turnover Intention

Antecedents in previous studies view customer incivility as low-intensity deviant customer behaviors put into action with the intention to harm or disrupt the shared norm for mutual respect [66], which are difficult for companies to react to since they are usually not illegal [5]. These rude actions that are prevalent and frequent in the service-oriented organizations have affected employees as a social and as a workplace stressor and have caused employees to feel a higher level of emotional exhaustion [32,67], job burnout [68], and a low level of job service performance [69].

The negative emotions and effects that customer incivility has on employees are the effects that are accused of having a profound effect on the employees’ turnover intention [9,13,36,67,68,69,70]. Along with the proof that is provided in the study by Han et al. [57], the following hypothesis is postulated.

**Hypothesis** **(H1).** *There is a positive relationship between customer incivility and employees’ turnover intention.*

#### 2.5.2. Job Stress and Turnover Intention

Job stress is referred to as a condition of stress that is derived from the characteristics of an individual’s job, such as work tasks, work overloads, or the workplace [39,40] and it has been the focus of a lot of scholarly attention in many different fields [45,50], which includes the hospitality industry. Tongchaiprasit and Ariyabuddhiphongs [71] studied the mediating effects of job stress in regard to turnover intention among hotel chefs, and Park, Kim, Hai and Dong [43] explored the detrimental effects of job stress on turnover intention. In light of the preceding findings, the following hypothesis is proposed.

**Hypothesis** **(H2).** *There is a positive relationship between the employees’ job stress and their turnover intention.*

#### 2.5.3. Social Support and Turnover Intention

Social support is the assistance one person could receive from interpersonal relationships which include both instrumental and emotional support [58]. Instrumental support from supervisors and co-workers such as sharing work-related information or providing feedback [58,65] is one of the necessities in the workplace but perceived emotional support has received more scholarly attention by proving that it has a positive influence on employee job satisfaction, job/work engagement, and organizational commitment which can reduce the employee turnover intention [59,72,73,74]. In line with the previous antecedents, the following hypotheses were constructed.

**Hypothesis** **(H3).** *There is a negative relationship between the employees’ perceived supervisor support and their turnover intention.*

**Hypothesis** **(H4).** *There is a negative relationship between the employees’ perceived co-worker support and their turnover intention.*

#### 2.5.4. Demographics and turnover intention

The demographic factors have also proven to have significant correlations with turnover intention. In Lambert’s study [75], women expressed turnover intention more than men. Additionally, the desire to leave decreased as the employees’ tenure increased. This finding is supported by other studies [76,77,78], which indicate that there are certain demographic factors that could affect turnover intention. Through the validation of the prior studies, this study aimed to examine the influence of demographic variables on turnover intentions and the variability. Hence, Hypotheses 5a–d were formulated as follows.

**Hypothesis** **(H5a).** *The turnover intention is influenced by gender.*

**Hypothesis** **(H5b).** *The turnover intention is influenced by marital status.*

**Hypothesis** **(H5c).** *The turnover intention is influenced by job positions.*

**Hypothesis** **(H5d).** *The turnover intention is influenced by years of service.*

## 3. Methodology

### 3.1. Measurement Items

In order to fulfill the objectives of the research, a questionnaire was drawn from the previous studies [69,73,79,80], which measured the five variants. To measure the level of customer incivility that the frontline employees felt, five items were adopted from the research of Cho, Bonn, Han, and Lee [69], which included “the customers took out their anger on me” and “the customers made insulting comments to me”.. The job stress that the employees felt at work was measured using four items, which were adapted from the research of Parker and Decotiis [79] studying the stressors that could arouse first-level outcome “job stress”. Some included items such as “I have too much work and too little time to do it” and “sometimes when I think about my job, I get a tight feeling in my chest”. Both the perceived supervisor support and the perceived co-worker support of the frontline employees were measured using the items that were used in the previous study by Tsui, Pearce, Porter, and Tripoli [80] (six items (e.g., “My supervisor is friendly”, “There is no ‘team spirit’ in my group”) were removed because their factor loadings were lower than 0.30 or not significant). There were three items for each construct. The employees were asked items that included “my supervisor seems willing to listen to my problems,” “my supervisor is considerate of subordinates’ feelings,” “we have confidence in one another in this group,” and “members of my workgroup show a great deal of integrity”. The employees’ desire to leave the current workplace was measured using three items from the study of Karatepe Osman [73], which include “I often think about quitting” and “I will probably quit this job next year”.

Demographic variables such as gender, marital status, years of service, and position were also measured in the following manner. The gender was coded with male = 1 and female = 2. The marital status was coded with *married* = 1 and *not married* = 2. The years of service was coded as follows: less than 2 years = 1, 2-5 years = 2, 5-10 years = 3, and over 10 years = 4. The positions were coded as follows: staff = 1, team leader/captain = 2, and others = 3. All the scale items were measured using a seven-point Likert’s scale that ranged from 1 (strongly disagree) to 7 (strongly agree), as indicated in the Appendix A. The construct reliability of all the scales was above 0.7.

### 3.2. Data Collection and Samples

The questionnaire was first prepared in English, and it was translated to Korean using the general algorithm of back-translation [81], and then reviewed by an academic professional in the hospitality industry before distribution in order to ensure the validity and reliability of the construct. With the finalized questionnaire, a pre-test was conducted on a sample that consisted of 11 undergraduates and graduate students in the field of hospitality at Sejong University to test the contents. They were asked to look over the questionnaires and give any feedback that is necessary.

By using a convenience sampling approach, the data was collected from a sample of frontline employees currently working at five-star hotels in Seoul, Korea using both the web and paper. When distributing the surveys, the research project and the objectives were explained thoroughly to all respondents. All of the participants were informed that their responses to the questionnaire would be kept confidential, and they would be used for research purposes only. From the end of 2019 until February of 2020, a total of 350 surveys were distributed, and 302 were returned as completed, which produced a response rate of 86.2%.

The demographic characteristics of the 302 participants were analyzed using frequency analysis, which is shown in Table 1. Of the 302 participants, 55% (*n* = 166) were female employees, whereas 45% (*n* = 136) were male employees. In regard to the marital status, 64.9% (*n* = 196) of the respondents were married, which is a significantly higher proportion than the 35.1% (106) of the respondents that indicated that they were single. The highest proportion of respondents indicated that they worked in the Front Office (59.3%), which was followed by Food and Beverages (37.7%), and others, such as Sales and Marketing (3%). In total, 59.9% (*n* = 181) of the participants indicated that they have a university degree, which was followed by 25.2% (*n* = 76) indicating that they held a two-year/community college degree, 13.9% (*n* = 42) stipulated that they have a graduate degree, and only 1% (*n* = 33) indicated that they held a high-school degree or less. A total of 69.2% (*n* = 209) of the respondents were regular employees, whereas 30.8% (*n* = 93) were contract employees. In regard to the years of service, 50% of the respondents indicated 2–5 years, which was followed by 10 years or more (30.5%), less than 2 years (12.9%), and 6–10 years (6.6%). Regarding the job position, staff positions ranked the highest at 50.7%, which was followed by team leader positions at 27.2%, and 22.2% indicated others, which included any job positions that are higher than the team leader’s position. Lastly, the participants’ annual income was asked. In total, 32.8% reported an income in the range of 25–39.99 million Korean Won, which was followed by an income that was 24.99 million Korean Won or less (30.5%), between 40 and 54.99 million Korean Won (26.8%), between 55 and 69.99 million Korean Won (7.9%), and 70 million Korean Won or higher (2%).

## 4. Results

### 4.1. Assessment of Reliability and Validity

The collected data from the finalized survey were screened to ensure only the valid respondents were subjects for the data analysis as well as to assess any violations of the analysis. To confirm the hypotheses presented in the study, SPSS 24.0 for Windows was used for the analysis. To confirm the reliability and the validity of the survey’s constructs, a reliability test was performed using the coefficient alpha to assess the internal consistency. Even though the reliability values above 0.70 are considered adequate, the minimum of standards is above 0.80, which is recommended by Clark and Watson [82] and Nunnally [83]. The Cronbach’s α of the variables in this study ranged from 0.892 to 0.912. Table 2 illustrates that these multiple items are highly reliable to measure each construct (Customer Incivility, 0.912; Job Stress, 0.892; Turnover Intention, 0.896; Perceived Supervisor Support, 0.897; Perceived Co-worker Support, 0.905). This shows that all values are greater than 0.500 which is considered to be an acceptable cut-off for reliability [83].

The measurement items were extracted from the empirical studies then adapted to fit the context of the current research. The Eigenvalue was adopted as a criterion to select the questions, which focused on extracting factors that can explain more than 1.0. The item could only be included in the final factor structure with Eigenvalue greater than 1 and factor loadings with covariance greater than 0.400 [84]. Therefore, after excluding items that were not sufficiently standard, further analyses were conducted on the 18 items ultimately filtered out. The results of the analysis indicated that the Kaiser–Meyer–Olkin (KMO) measure of sampling adequacy was 85.9%, which was above the criterion of 0.600 [85], thus, the results of the factor analysis of the model were considered acceptable. In addition, after the analysis, the items that had factor loads under 0.3 were excluded from the scale, which left a total of 18 questions to be analyzed. The data set was considered to be appropriate with the support of Kaiser–Meyer–Olkin (KMO) value of 0.859, which a value higher than 0.5 indicates supportive. All the factor loadings and communality were statistically significant and were greater than 0.6, which indicated the existence of convergence validity in the data. The AVE (average variance extracted) of the five factors, which is another criterion to test the validity, showed a range that was between 0.636 and 0.803. All the constructs displayed AVE values that were higher than 0.5, which supported the convergent validity of the constructs [86,87]. The reliabilities for the items and the result of the factor analysis are presented in Table 2.

The means, standard deviations, and correlations of the study variables are presented in Table 3. As reflected in the corresponding correlation coefficients, the fit of constrained models, which the correlations between the constructs were set to equal 1, and their unconstrained counterparts, which are correlations between the constructs that vary, for the 10 pairs associated with the five constructs. In support of the discriminant validity, each chi-square difference was significant at *p* < 0.01 [88].

### 4.2. Regression Analysis and Hypothesis Testing

The results of the multiple regression analyses used to test the hypotheses in the study are presented in Table 4. Findings from the analysis of employees’ turnover intention as the dependent variable showed that R square was 26.8% and adjusted R square was 25.8%. The overall F-value was 27.180, which significantly supports the model fit at the 1% statistic level (*p* < 0.000). In addition, the analysis yielded Durbin–Watson (DW) statistic of 2.212. Thus, it was able to indicate that there was no first-order autocorrelation in the residual term of the data in this study. The details of the regression analysis outcome for the hypotheses in this study are as follows. Hypothesis 1 suggested that there would be a positive relationship between customer incivility and the employee’s turnover intention, and Hypothesis 4 suggested that there would be a negative relationship between the perceived supervisor support and the employee’s turnover intention. However, both results showed that (customer incivility → turnover intention, β = 0.061, t = 0.992; perceived co-worker support → turnover intention, β = −0.006, and t = −0.107) the impact of customer incivility and the perceived co-worker support were insignificant. Therefore, Hypothesis 1 and Hypothesis 4 were rejected.

Hypothesis 2 suggested that there would be a positive relationship between job stress and the employee’s turnover intention. Job stress was found to be positive predictor of turnover intention (β = 0.244, t = 3.858, and *p* < 0.001) as predicted. Thus, Hypothesis 2 was supported. Additionally, as Hypothesis 3 predicted, the significant t-value (β = −0.345, t = −5.960, and *p* < 0.001) indicates that the perceived supervisor support impacts the employee’s turnover intention negatively. Therefore, Hypothesis 3 was supported.

### 4.3. Crosstabulation of Demographic Factors and Turnover Intention

Among the demographic background variables, the marital status and the job position were the variables that showed significant effects on the employee’s turnover intention. A high percentage of married respondents responded that they do not have the intention to leave their perspective organizations or plan on quitting their jobs (χ2 = 40.428 and ** *p* < 0.01). Additionally, a high percentage of the staff answered that they have the intention to leave their perspective organizations or plan on quitting their jobs compared to the respondents in the higher job positions (χ2 = 57.799 and * *p* < 0.05). Gender and years of service had no significant effects on turnover intention. Thus, Hypotheses 5b, c were supported. The crosstabulation is provided in Table 5.

## 5. Discussion

Due to the importance of retaining skillful, customer-oriented FLEs to maintain consistency in the service and products they provide [8], the study aims to explicate what influences the FLEs’ turnover intention, especially in the hotel industry. Four variables, which included customer incivility, job stress, perceived supervisor support, perceived co-worker support were selected as main variables from previous studies that consist of theories such as job–demands–resources theory or spiral of work incivility theory stating the importance of job resources in relation with job stress and work incivility within an organization [41,65]. Not only that, many previous studies have validated the existence of significant differences in demographic variables [75,76,77]. Therefore, building on the results of these previous studies, this study examined the differences between gender, marital status, job position, and years of service on turnover intention by using crosstabulation analysis. Two of the four hypothesized relationships linking the study variables including Hypotheses 2 and 3 were supported, which indicated that job stress has a significant influence on increasing turnover intention, and it showed that the perceived supervisor support had a negative relationship with the turnover intention of the employees. Hypotheses 1 and 4, which included customer incivility and perceived co-worker support, did not significantly influence turnover intention directly.

### 5.1. Theoretical Implication

The research findings of the study provide several theoretical implications in order to understand the employees’ turnover intention. A considerable number of studies have explored the relations among the variables over the past few decades [32,39,64,67], but the results vary considerably, which even show both positive and negative correlations between the same variables [13], and scholarly attempts to explore the direct influences on turnover intention receded, which make this empirical study more meaningful.

Firstly, customer incivility did not show a direct effect on the employees’ turnover intention when job stress proved to be a compelling reason for the employees to think of leaving their jobs. This result corroborates the previous studies where customer incivility was proven to be a variable that influenced factors, such as the employees’ job stress, emotional exhaustion, and job burnout [57,67,68,89], which in turn influences turnover intention [9,32]. The reason why a mediator is usually necessary in order to explain the positive relationship between customer incivility and turnover intention is unknown, but it can be argued that customer incivility is widely expected in the service industry, which confronts the actions itself and does not harm the employees in any way. Additionally, it can be thought that in many cases several hotel properties now place some kinds of disadvantages to those customers who have shown uncivil actions that are severe enough to damage the employee either physically or psychologically, such as no compensation, blocking further reservations, and implementing lawsuits. However, having to face these serious and minor incivilities constantly will surely influence the employees’ state of being [32], which can eventually affect the decision-making process regarding an employee quitting their job.

Secondly, in the research by Park and Min [13], supervisor support was found not to play a significant role in the hotel industry, which it did in other industries, and it was stated that the characteristics of hospitality jobs, which are labor-intensive, less complex, and need a minimum level of supervision, are one of the reasons. Additionally, for foreign workers, the perceived co-worker support was more proximal than the supervisor support, which resulted in less psychological stress [72]. De Clercq, Azeem, Haq, and Bouckenooghe [90] pointed out that the important reason co-worker support helps lower turnover intention is because it lowers the stress that the employees perceive in the work process. However, the perceived co-worker support was found to have no significant role that influenced turnover intention in this study. Additionally, the results from the present study provided considerable insights regarding the importance of the perceived supervisor support to reduce the turnover intention, which is in line with the existing research that explains that the supervisor support is an important factor regarding the employees’ job satisfaction [58], health, well-being at work [91], and quality of life [92]. The employees’ perceived supervisor support can determine whether their work effort is considered and cared about [58]. In other words, recognition and positive emotions the supervisors show to the employees can enhance their self-confidence, which in turn increases the workplace commitment. In the meantime, the effect of receiving recognition, praise, and support from co-workers can be less than that of the supervisors because the co-workers are at the same organizational level [72]. The perceived co-worker support can also be diluted because there are usually several co-workers at the same workplace, and some co-workers will be supportive, and others will not be. The perceived supervisor support can have a stronger effect on the level of support employees perceive since it usually comes from one important person who is in charge of direct supervision.

Lastly, the earlier studies [75,77] concluded that gender and tenure are determining factors for turnover intention. The higher turnover intention was found from the female employees, and as the employees’ number of working years increased, their turnover intention decreased. However, neither gender nor the years of service affected turnover intention in this research. This result can implicate several changes in society. One can argue that because society is more competitive, the motivation for a higher job position is more important than having safe long working years. Additionally, the conventional perception of a life-time job/workplace has faded over the years, which could urge the employees to change their workplace even after long working years. The result can also implicate that women are more active with their career development than in the past. These changes and their influence on employee behavior and the outcomes are all open for future research.

### 5.2. Practical Implication

The study also provides practical implications for real work situation practices. It was found that customer incivility did not have a direct effect on the turnover intention, but the job stress did. Therefore, hotel entrepreneurs should realize the importance of creating the proper environment for their employees as opposed to finding the problems from outside factors. One of the measurement items regarding job stress represents the failure of time management in a hotel. Being able to manage the staff appropriately is one of the key elements of management in every field, which has mostly been emphasized in the context of cost management [93]. However, it is important to approach the issue from the employees’ side because it can bring detrimental effects to job stress and, consequently, to the turnover intention of the employees.

Additionally, the hotel industry has the issue of seasonal variation, which is similar to most of the tourism industry. There are times in the hotel when the workload becomes overloaded up to the point where employees cannot manage the stress and frustration. Considering the characteristics of the hotel industry, it is inevitable that the number of visitors increases during certain seasons or during holidays. The dramatic change in the amount of work can certainly arouse the amount of stress the employees perceive. Therefore, creating adequate methods to efficiently staff during these certain times is also a definite role that organizations have to endure.

Since job stress and the perceived supervisor support were significant in influencing the employees’ intent to turnover, it can be assumed that there is a close relationship between job stress and perceived supervisor support. Thus, a way that hotels can lower job stress is by emphasizing the role of the supervisors. Supervisors can support their employees both instrumentally and emotionally [58]. Instrumental help is somewhat obvious and necessary, but training supervisors about the importance of emotional support and how they can be more supportive of their workers can help promote the employees’ perceived supervisor support, which will lower turnover intention.

Additionally, because the emotional supervisor support is helpful not only with individual levels but at team levels [58], the shared perception of supervisor support can enhance the satisfaction employees have toward the workplace and their job, and it can also stimulate the feeling of kinship between the team members grouping themselves as one team. Even though co-worker support did not play a significant role in turnover intention directly in this study, previous studies stated that co-worker support can be an important aspect of the work conditions as one of the positive job resources [13]. It is open for further research, but it would also be reasonable to think that higher co-worker support will influence employees in a positive way and also have a significant impact on turnover intention. Therefore, considering non-work-related events or sports activities that focus on enhancing fun among workers can be used to build keen relationships not only within co-workers but also between workers and supervisors.

The results regarding demographic variables can also be an implication that when hotels make retention strategies for their employees, it is important to make strategies that involve promoting talented and faithful employees because the job position is significantly correlated with turnover intention when years of service did not play an important role. Most of the staff answered that they have a higher intention to change jobs than those who had positions higher than staff. It could be possible because they are not satisfied with their current position at work, and they feel that they are not awarded enough or think a better opportunity lies elsewhere for their career development. Either way, the finding in this research that the staff with a higher position had more intentions to stay rather than the pervasive thought that they would be leaving due to better chances at different institutions with the help of sufficient work experience implicates the strong effect of the job position retaining employees.

### 5.3. Limitations and Future Research

Even though this study contributes to exploring the independent variables that influence the employee’s turnover intention, it is not free from limitations. First, the study’s sample was limited to frontline employees working at hotels in one geographic area. Thus, the representativeness of the study’s sampling is not sufficiently secured. Therefore, the findings of this study regarding the relationships between the variables should be considered with caution, not to be generalized since the prodigious discrepancy lies within the way people behave and react in different countries and different cultures. Additionally, the sample was restricted to the hotel employees who were working in five-star hotels only, and five-star hotel employees can be considered to have more work pressure due to the high-quality service demand that could influence the relationships between the variables. Extending the research to other regions and cultural contexts will improve the generalizability of the results. In addition, it would be interesting to see if there were any differences if the test was to be applied to smaller hotels that have lower quality service demand.

The present study explored the variables that include customer incivility, job stress, perceived supervisor support, and perceived co-worker support considering previous theoretical backgrounds such as spiral of work incivility theory [65]. However, there can be many decisive reasons that are plausible to motivate employees to continue working in a different environment or even change their occupational careers, such as better work opportunities.

The dependent variable used in the study was the turnover intention. Turnover intention is the best predictor for the actual turnover rate [9,75], but it is just a forecast, which means there will be an actual gap between the intention and the actual behavior. Additionally, even though the measurement items were extracted based on the previous studies, there were many items removed in the present research.

Extending the current theoretical framework by determining the other possible critical factors that could affect employee turnover, setting up the correct concept of variables, and exploring the correct measurement items for each variable might be fraught with methodological difficulties, but these types of research would help to provide valuable insights to the industry. Further studies can also be expanded to find if there is any variable that acts as a mediator between the relationships that were not significant in the present study in order to provide a better understanding of the field.

## 6. Conclusions

The turnover intention of employees, especially in those firms that are service-oriented, has been receiving a lot of scholarly attention. Businesses have become more competitive, and they are constantly facing changes that are both positive and negative, which have also influenced the previous research. Through this study, the purpose has remained to explore the influences the selected variables had on turnover intention. Results showed that job stress had a significant influence on increasing turnover intention and the perceived supervisor support had a negative relationship with the turnover intention of the employees. Regardless of the results drawn from the study, both the theoretical and empirical studies are in need of a sufficient understanding of the employees’ behavior. This study hopes to be an impetus for continuous studies on the employees’ psychological well-being, which in turn shed light on the development of the employee commitment and reduce negative work outcomes, such as turnover intention, which thereby make both the companies and the employees mutually beneficial.

## Figures and Tables

**Table 1:** Demographic characteristics.

Characteristics		Number	Percentage (%)
Gender	Male	136	45
	Female	166	55
Marital status	Single	196	64.9
	Married	106	35.1
Education	High school degree or less	3	1
	2 year/community college degree	76	25.2
	University degree	181	59.9
	Graduate degree	42	13.9
Work area	Regular employee	209	69.2
	Contract employee	93	30.8
Years of service	2 years or less	39	12.9
	2–5 years	151	50
	5–10 years	20	6.6
	10 years or more	92	30.5
Yearly income	KRW 24.99 million or less	92	30.5
	KRW 25–39.99 million	99	32.8
	KRW 40–54.99 million	81	26.8
	KRW 55–69.99 million	24	7.9
	KRW 70 million or more	6	2
Job position	Staff	153	50.7
	Team leader	82	27.2
	Others	67	22.2
Working unit	Front office	179	59.3
	F&B	114	37.7
	Others	9	3
Total		302	100%

**Table 2:** Results of the factor and reliability analyses.

Factors	Variable	Factor Loading	Communality	Eigenvalue	AVE	Cronbach α
Customer Incivility	CI1	0.875	0.823	36.736	0.685	0.912
	CI2	0.881	0.841			
	CI3	0.832	0.755			
	CI4	0.807	0.729			
	CI5	0.733	0.629			
Job Stress	JS1	0.847	0.870	18.575	0.636	0.892
	JS2	0.825	0.790			
	JS3	0.792	0.791			
	JS4	0.720	0.646			
Turnover Intention	TI1	0.758	0.754	6.737	0.710	0.896
	TI2	0.911	0.890			
	TI3	0.912	0.877			
Perceived Supervisor Support	PSS1	0.860	0.840	10.712	0.726	0.897
	PSS2	0.873	0.859			
	PSS3	0.822	0.785			
Perceived Co-worker Support	PCWS1	0.886	0.829	7.262	0.803	0.905
	PCWS2	0.927	0.888			
	PCWS3	0.875	0.808			
KMO = 0.859 Bartlett = 4084.954 df = 153 Sig = 0.000						

Notes. CI: customer incivility, JS: job stress, TI: turnover intention, PSS: perceived supervisor support, PCWS: perceived co-worker support. AVE: average variance extracted. KMO: Kaiser–Meyer–Olkin measure of sampling adequacy.

**Table 3:** Correlations and descriptive statistics.

Construct	[1]	[2]	[3]	[4]	[5]	Mean	SD
[1] CI	1					3.78	1.614
[2] JS	0.584 **	1				4.26	1.621
[3] TI	0.283 **	0.396 **	1			3.93	1.865
[4] PSS	−0.229 **	−0.335 **	−0.443 **	1		4.60	1.503
[5] PCWS	−0.033	−0.079	−0170 **	0.414	1	5.23	1.157

Notes. CI: customer incivility, JS: job stress, TI: turnover intention, PSS: perceived supervisor support, PCWS: perceived co-worker support. ** *p* < 0.01.

**Table 4:** Results of regression analysis.

	Turnover Intention			
	Unstandardized Coefficients		Standard Coefficients	t-Value
	B	S. E	β	
Customer Incivility	0.070	0.071	0.061	0.992
Job Stress	0.281	0.073	0.244	3.858 ***
Perceived Supervisor Support	−0.428	0.072	−0.345	−5.960 ***
Perceived Co-worker Support	−0.009	0.088	−0.006	−0.107
R^2^ = 0.268, Adjusted R^2^ = 0.258, F = 27.180 ***, DW= 2.212				

Note. S. E: standard error, DW: Durbin–Watson. *** *p* < 0.001.

**Table 5:** The crosstabulation of demographic factors.

	Turnover Intention							
	1	2	3	4	5	6	7	Total
Gender. χ^2^ = 15.541, *p* > 0.05								
Male	23	27	23	17	17	13	16	136
Female	13	22	26	28	33	23	21	166
Total	36	49	49	45	50	36	37	302
Marital Status. χ^2^ = 40.428, ** *p* < 0.01								
Single	18	23	34	36	34	20	31	196
Married	18	26	15	9	16	16	6	106
Total	36	49	49	45	50	36	37	302
Job Position. χ^2^ = 57.799, * *p* < 0.05								
Staff	12	17	26	31	27	16	24	153
Team leader/captain	12	21	12	8	13	11	5	82
Others	12	11	11	6	10	9	8	67
Total	36	49	49	45	50	36	37	302
Years of Service. χ^2^ = 58.711, *p* > 0.05								
Less than 2 years	4	3	5	10	6	2	9	39
2–5 years	15	20	25	23	28	21	19	151
5–10 years	0	3	5	2	2	4	4	20
10 years or more	17	23	14	10	14	9	5	92
Total	36	49	49	45	50	36	37	302

Note 1. 1 = “strongly disagree”, 2 = “disagree”, 3 = “somewhat disagree”, 4 = “neither agree nor disagree”, 5 = “somewhat agree”, 6 = “agree”, 7 = “strongly agree”. * *p* < 0.05 and ** *p* < 0.01.

## Data Availability

The dataset used in this research is available upon request from the corresponding author. The data are not publicly available due to restrictions i.e., privacy or ethical.

## Appendix A

Customer Incivility—(Cho, Bonn, Han, and Lee, 2016)

Customers took out their anger on me.

Customers made insulting comments on me.

Customers treat employees as if they were inferior.

Customers showed that they are impatient.

Customers do not trust the information that I gave them and ask to speak someone of higher authority.

Job Stress—(Parker and Decotiis, 1983)

My job gets to me more than it should.

I have too much work and too little time to do it in.

There are lots of times when my job drives me right up the wall.

Sometimes when I think about my job, I get a tight feeling in my chest.

Perceived Supervisor Support—(Tsui, Pearce, Porter, and Tripoli, 1997)

My supervisor seems willing to listen to my problems.

My supervisor is considerate of subordinate’s feelings.

I can rely on my supervisor.

Perceived Co-worker Support—(Tsui, Pearce, Porter, and Tripoli, 1997)

We are usually considerate of one another’s feelings in this work group.

We have confidence in one another in this group.

Members of my work group show a great deal of integrity.

Turnover Intention—(Karatepe Osman, 2013)

It is likely that I will actively look for a new job next year.

I often think about quitting.

I will probably quit this job next year.
